# Erector spinae plane block with bupivacaine contributes to intraoperative opioid sparing but provides limited postoperative pain control in cats undergoing elective ovariohysterectomy under continuous propofol infusion

**DOI:** 10.1007/s11259-026-11180-w

**Published:** 2026-03-28

**Authors:** Ana Paula Longo Ribeiro, Gilberto Serighelli-Júnior, Felipe Comassetto, Lorenzo Schmitz Borsato Cavagnari, Camila Fernanda Baehr Cavagnari, Atila Souza Rocha Freire de Santana, Maryana de Souza Matos, Ayla da Costa Wittaczik, Nilson Oleskovicz

**Affiliations:** 1 Department of Veterinary Medicine, Center for Agroveterinary Sciences, Santa Catarina State University, Lages, Brazil; 2 Department of Veterinary Medicine, Federal University of Paraná, Curitiba, PR Brazil

**Keywords:** Acute pain, Erector spinae plane block, Abdominal analgesia, Cats, Opioid consumption

## Abstract

This study evaluated the intraoperative and postoperative analgesic effects of an ultrasound-guided erector spinae plane (ESP) block with bupivacaine in cats undergoing elective ovariohysterectomy. Sixteen healthy client-owned female cats (with, 2.62 ± 0.46 kg; and, 23.06 ± 20.34 months) were randomly assigned to two groups (*n* = 8 each). All animals received dexmedetomidine (2.5 µg/kg, intramuscularly), followed by propofol administered to effect for induction and maintained as a continuous rate infusion beginning at 0.3 mg/kg/min adjusted as necessary. Ultrasound-guided bilateral ESP blocks were performed at the first lumbar vertebra using 0.25% bupivacaine (0.5 mL/kg per side) in the bupivacaine group, (GB) or 0.9% saline in the saline group (GS). Intraoperative Cardiopulmonary variables and nociceptive responses were recorded, and fentanyl (2.5 µg/kg, IV) was administered as analgesia rescue. Postoperative pain was assessed over 24 h using the UNESP–Botucatu Multidimensional Pain Scale (short-form) and the Feline Grimace Scale, with buprenorphine rescue (20 µg/kg, IM). Cats in GB required fewer intraoperative fentanyl rescue (27 vs. 35 administrations), corresponding to a 22.85% reduction in total fentanyl rescue compared with the GS, however, no statistical difference between groups (*p* = 0.5976). Intraoperative heart rate and systolic arterial pressure increased during periods of greater surgical stimulation in both groups, with no significant between-group differences. No significant differences were observed in postoperative requirement of analgesic rescue. (*p* = 0.9554). These findings indicate that the ESP block with bupivacaine provides effective intraoperative opioid-sparing analgesia but limited postoperative benefit, supporting its use as part of a multimodal analgesic approach in feline abdominal surgery.

## Introduction

Ultrasound-guided fascial plane blocks have expanded the use of regional anesthesia by allowing accurate needle placement and controlled deposition of local anesthetics within fascial compartments (Elsharkawy et al., 2018). These techniques are intended to provide perioperative analgesia through blockade of neural structures located within or adjacent to fascial planes, potentially reducing systemic anesthetic and analgesic requirements (Chin and El-Boghdadly, 2021; Portela et al., 2021). Among these techniques, the erector spinae plane (ESP) block involves injection of local anesthetic dorsal to the transverse process and ventral to the erector spinae muscle group (Forero et al., 2016; Medina-Serra et al., 2021). Initially described in humans for the management of neuropathic thoracic pain (Forero et al., 2016), the ESP block has subsequently been evaluated in anatomical and clinical studies, demonstrating variable patterns of injectate spread and clinical applicability (Zannin et al., 2020; Medina-Serra et al., 2021). More recently, the ESP block has been investigated in veterinary patients undergoing abdominal surgery, where ultrasound-guided ESP injection was associated with reduced intraoperative opioid requirements in dogs undergoing ovariohysterectomy, although with limited effects on postoperative analgesia (Schmitz et al., 2026). The increasing interest in regional anesthetic techniques is partly driven by the need to reduce perioperative opioid use. Opioid administration is associated with several adverse effects in veterinary patients, including gastrointestinal, cardiorespiratory, and behavioral alterations (KuKanich and Wiese 2015; Didier et al. 2024). In human medicine, the development of opioid tolerance and opioid-induced hyperalgesia has been associated with escalating opioid requirements and an increased likelihood of prolonged opioid exposure (Bohringer et al. 2020; Thota et al. 2019). These concerns have contributed to a growing emphasis on opioid-sparing analgesic strategies, particularly in North America and Europe. In the United States, opioid misuse has reached epidemic levels, with a substantial increase in overdose deaths involving synthetic opioids reported over the past decade (Bohringer et al. 2020). Consequently, medically prescribed opioids are increasingly recognized as a significant iatrogenic factor contributing to the current opioid crisis (Bohringer et al. 2020; Thota et al. 2019). In this context, there has been growing interest in analgesic strategies that reduce perioperative opioid requirements. Regional anesthetic techniques have demonstrated the potential to attenuate neuroendocrine surgical stress responses, improve recovery quality, and reduce postoperative pain scores compared with systemic opioid administration, further supporting their clinical relevance (Romano et al., 2016).

The ESP block is a regional anesthetic technique of moderate to advanced complexity that can be applied at cervical, thoracic, and lumbar levels (Forero et al., 2016). Initially indicated for analgesia in spinal disorders, its use has progressively expanded to abdominal procedures in both human and veterinary medicine (Vidal et al., 2018; Alza Salvatierra et al., 2021; Gómez Fernández et al., 2021). In veterinary medicine, the ESP block has been reported as part of multimodal analgesia for thoracic and spinal surgeries (Bartholomew and Ferreira 2021; Portela et al., 2020; Viilmann et al., 2022). In dogs undergoing lateral thoracotomy, ultrasound-guided ESP block with bupivacaine, combined with epidural morphine, resulted in effective perioperative analgesia (Gómez Fernández et al., 2021). The ESP block has also been reported for analgesic management in a canine case of pancreatitis, in documenting clinically relevant pain control following the administration of 0.25% bupivacaine (Bartholomew and Ferreira 2021). Furthermore, retrospective studies evaluating dogs undergoing hemilaminectomy have shown that the use of the ESP block was associated with reduced perioperative analgesic consumption, particularly opioids, and a low incidence of complications (Portela et al., 2020; Viilmann et al., 2022).

This technique may also be applied within opioid-free or opioid-sparing anesthetic protocols. An example of its clinical applicability was reported by Zannin et al. (2020), who described opioid-free total intravenous anesthesia combined with ESP block in a dog undergoing hemilaminectomy. In addition, the use of regional techniques such as the ESP block may contribute to a reduction in perioperative opioid requirements. Opioid tolerance and opioid-induced hyperalgesia have been extensively described in human perioperative medicine and are associated with increased opioid requirements and a higher risk of long-term dependence (Bohringer et al. 2020; Thota et al. 2019). These effects have contributed to the implementation of alternative perioperative analgesic strategies aimed at reducing opioid use, particularly in North America and Europe, with increased emphasis on multimodal and regional techniques to minimize opioid exposure (Bohringer et al. 2020; Thota et al. 2019; Zannin et al. 2020). Comparable opioid-related adverse effects have also been reported in veterinary patients (KuKanich and Wiese 2015).

In cats, clinical evidence regarding the analgesic efficacy of the ESP block remains limited. A clinical study evaluating ultrasound-guided ESP block with bupivacaine in three cats undergoing spinal surgery suggested that this technique may be suitable as part of multimodal analgesia in felines (Alza Salvatierra et al., 2021). However, robust clinical evidence supporting its efficacy for providing visceral and somatic abdominal analgesia in cats undergoing ovariohysterectomy is still lacking, highlighting the need for controlled clinical investigations. Since its first description by Forero et al. (2016), the ESP block has been evaluated in several clinical and cadaveric studies. However, reported results have been inconsistent, particularly regarding injectate spread patterns and the extent of neural involvement, leading to uncertainty about the reproducibility and mechanisms underlying its analgesic effects.

Therefore, this study aimed evaluated the intraoperative and postoperative analgesic effects of an ultrasound-guided erector spinae plane (ESP) block with bupivacaine in cats undergoing elective ovariohysterectomy. It was hypothesized that cats receiving the ESP block would require fewer intraoperative and postoperative analgesic rescue interventions compared with a saline group not receiving local anesthetic injections.

## Materials and methods

### Animals

This prospective, double-blinded, randomized, placebo-controlled, parallel-group clinical trial was designed and reported in accordance with the CONSORT guidelines for randomized controlled trials. The study was conducted at Santa Catarina State University (UDESC) and approved by the Institutional Committee for the Ethical Use of Animals (CEUA) under protocol no. 7,959,250,624. Sixteen privately owned, mixed-breed female cats were enrolled, with a body weight of 2.62 ± 0.46 kg, mean age of 23.06 ± 20.34 months, and mean body condition score of 3.88 ± 0.83. Written informed consent was obtained from all owners prior to inclusion.

**Fig. 1:**
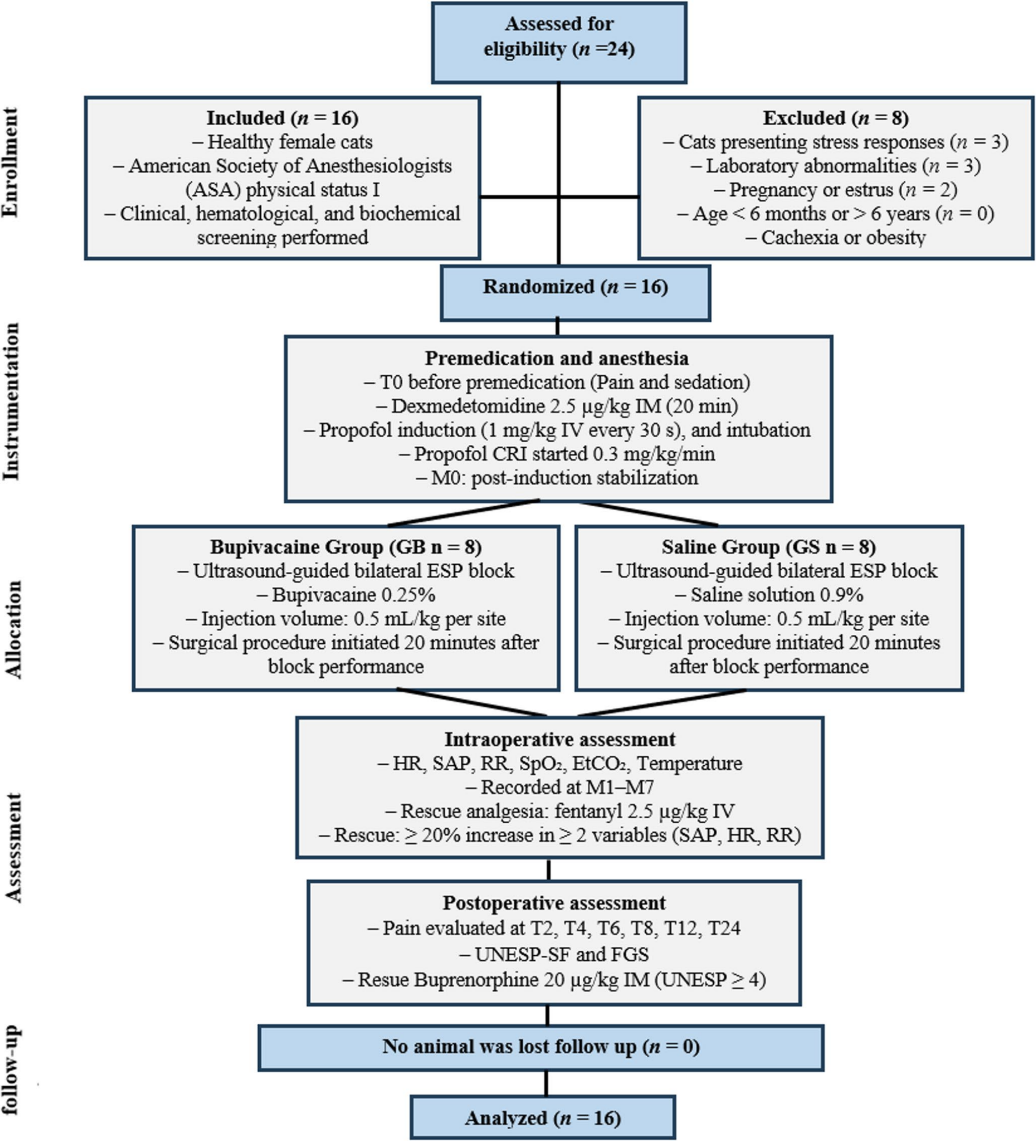
Flowchart illustrating study design, animal enrollment, randomization, anesthetic protocol, treatment allocation, intraoperative and postoperative assessments, and final analysis

Sample size estimation was performed using G*Power software (version 3.1.9.7; Erdfelder, Faul, and Buchner, Düsseldorf, Germany), assuming a two-sided α level of 0.05 and a statistical power (1–β) of 80%. The calculation followed a methodology similar to that reported by Junior et al. (2024), using the number of intraoperative fentanyl rescue boluses as the primary outcome variable. A 20% reduction in the number of fentanyl rescues was considered clinically relevant, with an estimated standard deviation of 5%. Based on these assumptions, a minimum sample size of eight animals per group was deemed sufficient to detect the hypothesized difference. Eligibility criteria included female cats classified as ASA I following health screening based on physical examination and laboratory evaluation. The preoperative diagnostic panel included complete blood count, urea, creatinine, alanine aminotransferase (ALT), alkaline phosphatase (ALP), albumin, total plasma protein, and total serum protein. Cats presenting comorbidities, cardiac disease, cachexia, obesity, aggressive behavior, laboratory abnormalities, confirmed pregnancy, estrus, or age below 6 months or above 6 years were excluded from the study (Fig. 1).

### Randomization

Cats were allocated to treatment groups using complete randomization generated by an online randomization tool *(*www.random.org*)* with a balanced 1:1 allocation ratio. Cats assigned to the bupivacaine group (GB: *n* = 8) received an erector spinae plane block at the level of the first lumbar vertebra (L1) using a transverse lumbar approach (Fig. 1), with injection of 0.25% bupivacaine (bupivacaine hydrochloride, 5.0 mg/mL; Cristália) at a volume of 0.5 mL/kg per injection site. Cats assigned to the saline group (GS: *n* = 8) received 0.9% saline (0.9% sodium chloride solution; 500 mL; Cristália) at an equivalent volume using the same technique. Investigators responsible for anesthetic management, intraoperative monitoring, and postoperative evaluations were blinded to group assignment throughout the study.

### Anesthesia instrumentation

To minimize stress, cats were housed individually before and after surgery, and all procedures were performed by the same surgeon. Food was withheld for 8 h and water for 2 h before anesthesia. Cats were admitted to the hospital 24 h prior to the experimental procedures for acclimatization and underwent shaving of the abdominal region, areas designated for block performance, and cephalic veins.

Prior to surgical preparation, cats were weighed using a digital scale, and baseline pain and sedation scores were recorded (T0) using the Feline Multiparametric Sedation Scale (Rutherford et al., 2022), the UNESP–Botucatu Multidimensional Pain Scale (short form) (Brondani et al., 2013), and the Feline Grimace Scale, as described by Evangelista et al. (2019). Following baseline assessment (T0), all animals received premedication with dexmedetomidine (2.5 µg/kg, IM) (Dexdomitor; 0.5 mg/mL; Zoetis). Sedation was reassessed 20 min after drug administration. Immediately after sedation reassessment, a cephalic vein was catheterized, and anesthetic induction was initiated. General anesthesia was induced with propofol (Propovan 10 mg/mL; Cristália) administered intravenously at a dose of 1 mg/kg every 30 s via hand injection, until adequate conditions for orotracheal intubation were achieved, defined by loss of jaw tone, absence of swallowing reflex, and reduction or absence of palpebral reflexes. Orotracheal intubation was performed using a Murphy-type endotracheal tube of appropriate diameter with a high-volume, low-pressure cuff. To facilitate intubation, 0.2 mL of lidocaine (Xylestesin^®^ 20 mg/mL; Cristália, Brazil) without vasoconstrictor was administered periglottically. The endotracheal tube was then connected to an anesthetic machine (Datex Ohmeda 9100c; GE Healthcare^®^) via a non-rebreathing Mapleson D anesthetic circuit, with delivery of 100% oxygen at a flow rate of 200 mL/kg/min.

Anesthesia was maintained with propofol administered as a continuous rate infusion (CRI) initiated at 0.3 mg/kg/min via a syringe pump (ST1000; Samtronic, Brazil). The infusion rate was adjusted by incremental increases or decreases of 25% relative to the initial rate to achieve and maintain an adequate anesthetic depth. After stabilization at anesthetic plane C (medium), as described by Ribeiro et al. (2009), characterized by absence of the palpebral reflex, ventral rotation of the globe, and preservation of the corneal reflex, the infusion rate was maintained accordingly. Lactated Ringer’s solution (Lactate Ringer 500 mL; Cristália, Brazil) was administered intravenously at a constant rate of 3 mL/kg/h throughout the surgical procedure.

## Ultrasound-guided block technique

**Fig. 2:**
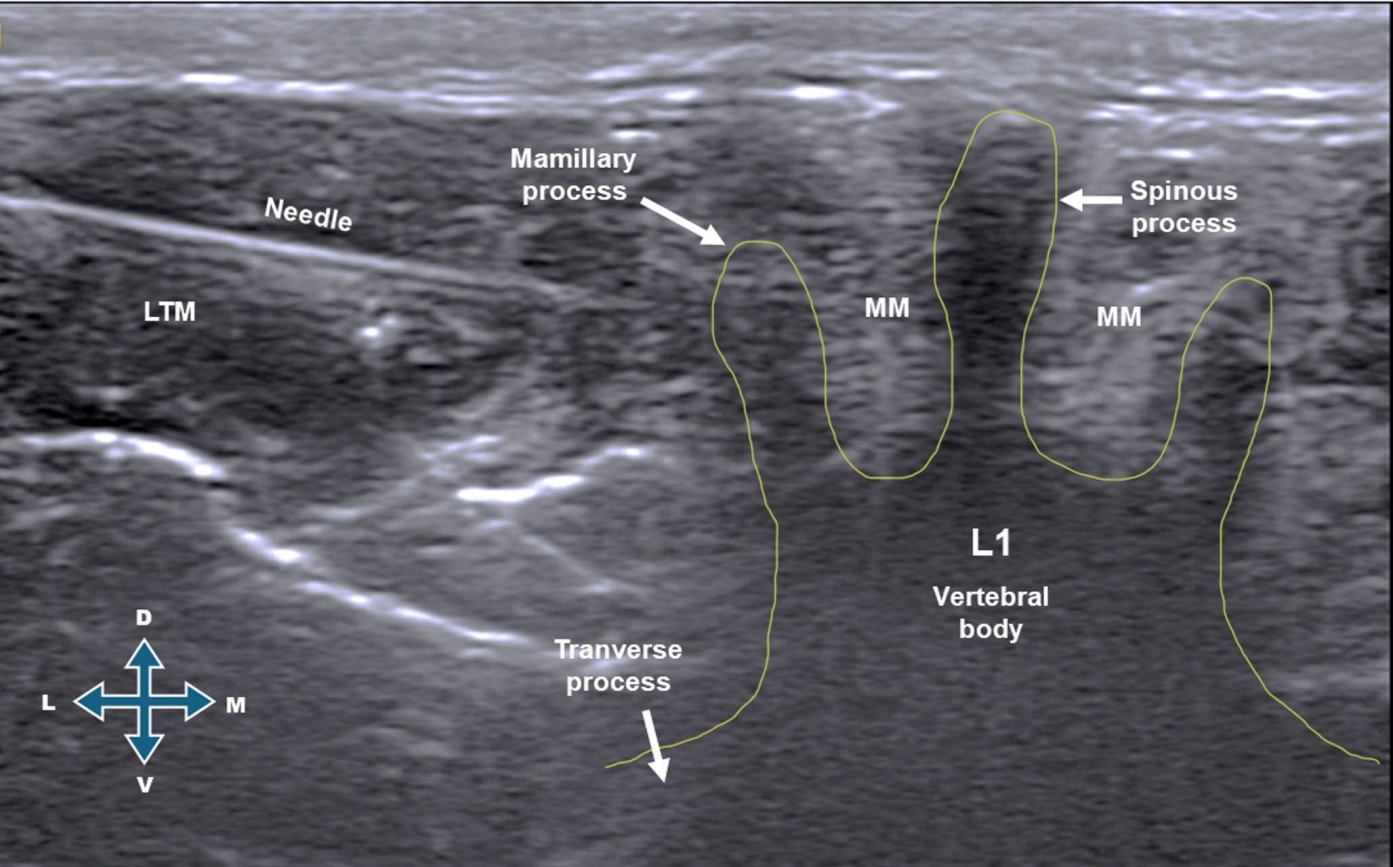
Transverse approach ultrasound-guided erector spinae plane (ESP) block at the L1 vertebral level in cats. Ultrasound image showing the needle (N) positioned at the target site for the ESP block, with the needle tip located on the mammillary process (MP), between the transverse process (TP) and the erector spinae muscle group. The multifidus muscle (MM) and longissimus thoracis muscle (LTM) are identified superficial to the vertebral structures. The vertebral body of the first lumbar vertebra (L1) is outlined. Anatomical orientation is indicated as medial (M), lateral (L), dorsal (D), and ventral (V)

After stabilization at anesthetic plane C, ultrasound-guided anesthetic blocks were performed by the same previously trained anesthesiologist using a linear L12-4 transducer (Lumify Linear Array Transducer; Philips Ultrasound, Inc., WA, USA). Cats allocated to the bupivacaine group (GB) received an erector spinae plane block at the level of the first lumbar vertebra (L1) using a transverse lumbar approach (Fig. 2), with injection of 0.25% bupivacaine (Bupivacaine Cloridrate; 5.0 mg/mL; Critália) at a volume of 0.5 mL/kg per injection site. Cats in the saline group (GS) received 0.9% saline (Saline solution 0.9%; 500 mL; Cristália) in an equivalent volume, administered using the same technique. Twenty minutes after injection at the second site, the ovariohysterectomy procedure was initiated.

### Experimental design

During surgery, rescue analgesia was administered when increases ≥ 20% relative to baseline values recorded after anesthetic stabilization were observed in at least two of the following variables: systolic arterial pressure (SAP), heart rate (HR), or respiratory rate (RR) (Conterno et al., 2025). In such cases, surgical stimulation was temporarily interrupted, and fentanyl (2.5 µg/kg, IV) was administered as a slow bolus over 1 min. Following bolus administration, SAP, HR, and RR were reassessed, and additional fentanyl boluses were administered as required until at least two variables returned to within 20% of baseline values. All rescue interventions were recorded.

Intraoperative monitoring included heart rate (HR, beats/min) assessed via electrocardiography, peripheral oxygen saturation (SpO₂, %) measured by pulse oximetry, respiratory rate (RR, breaths/min), end-tidal carbon dioxide concentration (EtCO₂, mmHg) measured using a capnograph connected to the anesthetic circuit, and esophageal temperature (°C). Systolic arterial pressure (mmHg) was measured using Doppler ultrasonography, with cuff size selected, corresponding to approximately 40% of the limb circumference.

Physiological variables were recorded at the following time points: M0 (after anesthetic induction and stabilization), M1 (5 min after completion of the ESP block), M2 (15 min after the block), M3 (skin incision), M4 (clamping of the right ovarian pedicle), M5 (clamping of the left ovarian pedicle), M6 (ligation of the cervix), and M7 (celiorrhaphy). The number and timing of intraoperative rescue analgesic interventions were also recorded. At the end of surgery, the propofol infusion was discontinued, and extubation and recovery times were recorded in minutes. Bradycardia and hypotension were treated with atropine (0.044 mg/kg, IV, Atropine sulfate, 0.25 mg/mL, Atrofarma, Brazil). In cases of hypotension, ephedrine (0.1 mg/kg, IV, Efedrina, 50 mg/mL, Cristália, Brazil) was administered initially; if systolic arterial pressure remained below 90 mmHg, a dopamine (Dopacris, 5 mg/mL Cristália, Brazil) continuous infusion was initiated at 5 µg/kg/min (IV).

### Postoperative pain and sedation assessment

Postoperative assessments were performed by the same experienced veterinary anesthetist, who was blinded to group allocation. Pain was evaluated immediately before premedication (T0) and at 2, 4, 6, 8, 12, and 24 h post-extubation using the UNESP–Botucatu Multidimensional Pain Scale (short form) (Brondani et al., 2013) and the Feline Grimace Scale (FGS), as described by Evangelista et al. (2019). Rescue analgesia was based exclusively on the UNESP–Botucatu scale (short form): cats with a total score ≥ 4 received buprenorphine (20 µg/kg, IM, Beniv, 0.04 mg/mL, Ourofino, Brazil), followed one hour later by dipyrone (25 mg/kg SC BID; Febrax 500 mg/mL, Lema, Brazil) and meloxicam (0.1 mg/kg, IM, Flamavet 0.2%; Agener, Brazil), after which the animal was removed from the study. Cats not requiring rescue analgesia were observed for 24 h postoperatively, after which nonsteroidal anti-inflammatory drugs were administered and the animals were discharged.

Sedation was assessed using the Feline Multiparametric Sedation Scale (FMSS; Rutherford et al., 2022) at the following time points: T0 (before premedication), after premedication 20 min, and at 2, 4, 6, and 8 h after extubation, allowing differentiation between sedative and analgesic effects on behavior.

### Statistical analysis

Statistical analyses were performed using GraphPad Prism^®^ (version 9.3, GraphPad Software Inc., USA), with a significance level set at *p* < 0.05. Data distribution was assessed using the Shapiro–Wilk normality test. Parametric data were analyzed using repeated-measures ANOVA with Dunnett’s test for comparisons with baseline and unpaired t-tests for between-group comparisons at each time point. Nonparametric data were analyzed using the Friedman test for repeated measures within groups, followed by Dunn’s test, and the Mann–Whitney test for between-group comparisons. Survival analysis for rescue analgesia was performed using Kaplan–Meier curves to estimate the probability of remaining free from additional analgesic intervention during both the intraoperative period (fentanyl) and the first 24 h postoperatively (buprenorphine). Data are presented as mean ± standard deviation or median (minimum–maximum), as appropriate.

## Results

Sixteen female cats were included in the study. No significant differences were observed between groups regarding age, body weight, or body condition score. The bupivacaine group (GB) had a mean age of 17.13 ± 17.63 months compared with 29.00 ± 21.51 months in the saline group (GS) (*p* = 0.2473). Mean body weight was 2.56 ± 0.34 kg in GB and 2.69 ± 0.55 kg in GS (*p* = 0.5831). Body condition score was 3.75 ± 0.89 in GB and 4.00 ± 0.53 in GS (*p* = 0.7582). Stabilization time was similar between GB (19 ± 6.5 min) and GS (18 ± 5.4 min), (*p* = 0.6825). Induction and intubation times were also comparable, with mean durations of 4.63 ± 1.19 min in GB and 3.75 ± 1.28 min in GS (p = *p* = 0.1812). Block performance time did not differ between groups, averaging 4 ± 1.5 min in GB and 4 ± 1.2 min in GS (*p* = 0.4813). Likewise, total anesthesia duration was similar between GB (77.5 ± 13 min) and GS (81.6 ± 14.7 min) (*p* = 0.5606), as was surgical time, which averaged 32.2 ± 7 min in GB and 37.1 ± 12.8 min in GS (*p* = 0.3588). Extubation time was also comparable between groups, averaging 17.75 ± 12.19 min in GB and 11.50 ± 7.80 min in GS (*p* = 0.2422).

**Fig. 3:**
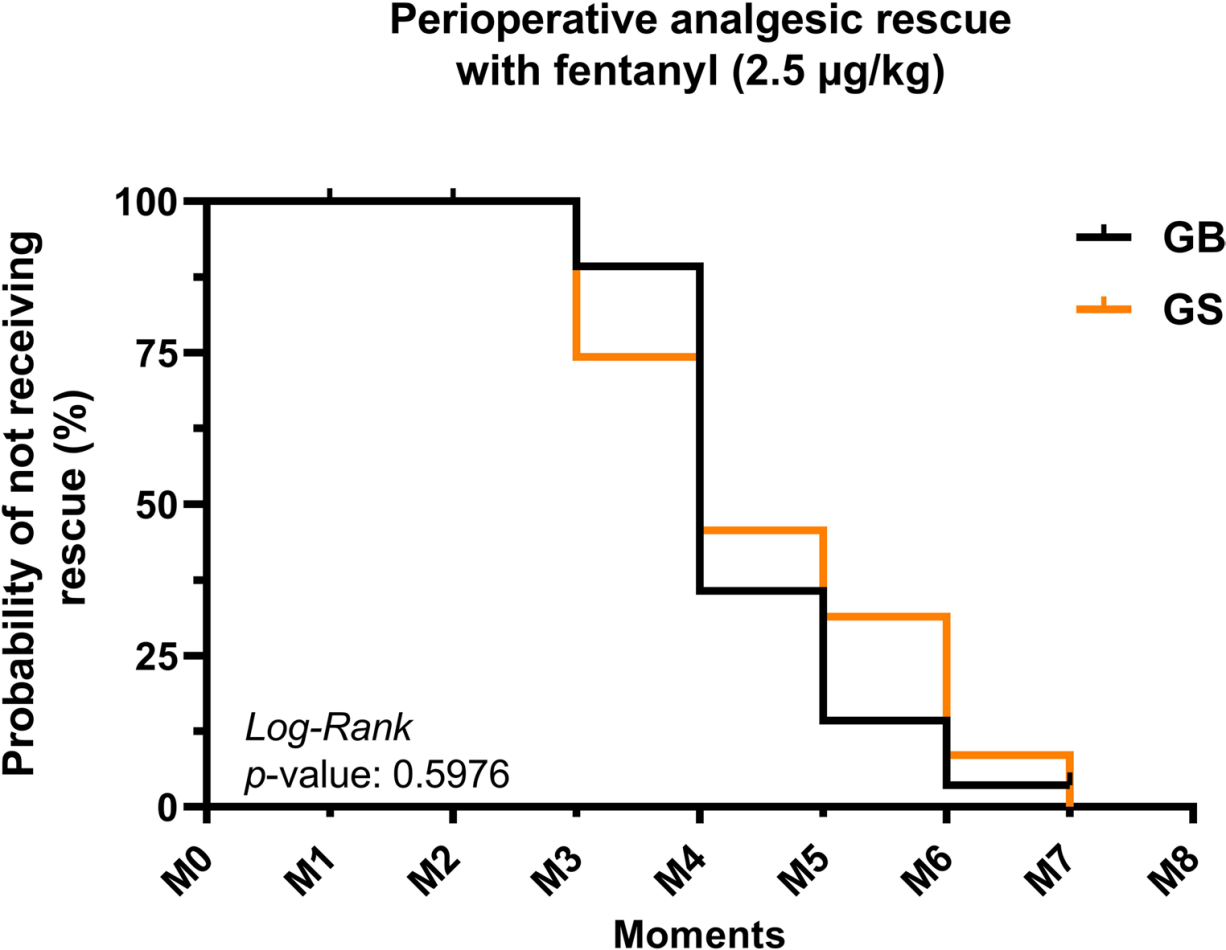
Kaplan–Meier curve illustrating the cumulative probability of remaining free from intraoperative fentanyl rescue (2.5 µg/kg) in cats anesthetized with propofol and undergoing elective ovariohysterectomy after an ultrasound-guided erector spinae plane block with 0.25% bupivacaine (GB) or saline solution (GS). No statistically significant difference was detected between groups (log-rank test, *p* = 0.5976)

The total number of intraoperative fentanyl rescue boluses was lower in the bupivacaine group (GB), with 27 administrations recorded, compared with 35 administrations in the GS (*p* = 0.5976). (Table 1). Kaplan–Meier analysis showed a progressive decrease in the probability of remaining free from intraoperative fentanyl rescue in both groups throughout surgery (Fig. 3). Although no statistically significant difference was detected between groups (log-rank, *p* = 0.5976), the GS exhibited an earlier decline in the survival curve, indicating a higher cumulative demand for rescue analgesia. Consistently, cats receiving the erector spinae plane block required fewer fentanyl boluses overall, corresponding to a 22.85% reduction in total fentanyl rescues, suggesting a clinically relevant intraoperative opioid-sparing trend despite the lack of statistical significance.

**Table 1:** Number of cats requiring fentanyl rescue analgesia (2.5 µg/kg) at each predefined surgical time point following an erector spinae plane block with 0.25% bupivacaine (GB) or saline solution (GS). Data are presented as the number of cats receiving fentanyl at each time point and the total number of fentanyl rescue boluses administered per surgical moment

Moments	Group	2.5µg/kg	5.0µg/kg	10µg/kg	Total number of rescues
M0	GS	0	0	0	0
	GB	0	0	0	0
M1	GS	0	0	0	0
	GB	0	0	0	0
M2	GS	0	0	0	0
	GB	0	0	0	0
M3	GS	3	3	0	9
	GB	3	0	0	3
M4	GS	4	3	0	10
	GB	1	7	0	15
M5	GS	1	2	0	5
	GB	4	1	0	6
M6	GS	2	1	1	8
	GB	1	1	0	3
M7	GS	1	1	0	3
	GB	0	0	0	0
Total	GS	11	10	1	35
	GB	9	9	0	27

Moments: M0 (after anesthetic induction and stabilization), M1 (5 min after completion of the ESP block), M2 (15 minutes after the block), M3 (skin incision), M4 (clamping of the right ovarian pedicle), M5 (clamping of the left ovarian pedicle), M6 (ligation of the cervix), and M7 (celiorrhaphy)

Heart rate (HR), systolic arterial pressure (SAP), respiratory rate (RR), end-tidal carbon dioxide (EtCO₂), and body temperature are summarized in Table 2. In both groups, HR increased from baseline during periods of greater surgical stimulation (M4–M6), reflecting a physiological response to nociceptive input, with no significant differences detected between groups at any time point. Similarly, SAP showed transient elevations during the same surgical stages, with a more sustained increase observed in the saline group at later time points. Bradycardia requiring atropine administration (0.044 mg/kg IV) was observed in two animals in the bupivacaine group at M7. Following atropine administration, heart rate returned to values similar to those observed at M0 (after anesthetic induction and stabilization) without inducing tachycardia, and no animals required vasopressor support. Respiratory rate and EtCO₂ exhibited time-dependent variations consistent with changes in surgical stimulation and anesthetic depth, while body temperature decreased progressively over the intraoperative period in both groups. No clinically relevant between-group differences were observed for respiratory or thermal variables.

The propofol infusion rate remained stable throughout the anesthetic period in both groups (Table 2). The mean infusion rate was 0.20 ± 0.10 mg/kg/min in the bupivacaine group (GB) and 0.24 ± 0.05 mg/kg/min in the saline group (GS) with no significant difference between groups (*p* = 0.4663). No significant changes over time were observed within either group (GB: *p* = 0.3471; GS: *p* = 0.1089). These findings indicate consistent maintenance of anesthetic depth according to predefined clinical criteria.

**Table 2:** Mean ± standard deviation of intraoperative physiological variables in cats undergoing elective ovariohysterectomy following an erector spinae plane block with 0.25% bupivacaine (GB) or saline solution (GS) administered at 0.5 mL/kg per injection site

	Group	HR	RR	SAP	SpO_2_	EtCO_2_	T^o^C	Propofol
M0	GS	112 ± 34	21 ± 7	105 ± 11	99 ± 1	26 ± 8	38.3 ± 0.8	0.22 ± 0.1
	GB	107 ± 21	21 ± 5	105 ± 12	100 ± 1	24 ± 7	38.4 ± 0.4	0.19 ± 0.09
M1	GS	104 ± 32	18 ± 5	108 ± 15	99 ± 3	28 ± 7	38 ± 0.7	0.24 ± 0.08
	GB	106 ± 12	23 ± 7	105 ± 10	98 ± 3	24 ± 5	38.2 ± 0.6	0.22 ± 0.1
M2	GS	112 ± 31	14 ± 5*§	106 ± 20	99 ± 1	29 ± 7	37.5 ± 0.7*	0.22 ± 0.09
	GB	100 ± 13	23 ± 5§	100 ± 7	99 ± 1	26 ± 6	37.9 ± 0.6	0.21 ± 0.08
M3	GS	140 ± 31	22 ± 7	129 ± 20	98 ± 2	29 ± 7	37.4 ± 0.9*	0.27 ± 0.1
	GB	125 ± 17	29 ± 9*	118 ± 14	99 ± 1	26 ± 7	37.7 ± 0.6*	0.24 ± 0.08
M4	GS	157 ± 30*	17 ± 7§	166 ± 36*	99 ± 1	34 ± 6	37.3 ± 0.9*	0.27 ± 0.1
	GB	156 ± 21*	29 ± 11§	159 ± 18*	99 ± 1	28 ± 7	37.6 ± 0.6*	0.24 ± 0.09
M5	GS	154 ± 25*	14 ± 4	151 ± 43*	99 ± 1	36 ± 6*	37.3 ± 0.9	0.26 ± 0.11
	GB	155 ± 24*	13 ± 4*	136 ± 20*	98 ± 2	33 ± 8*	37.5 ± 0.6*	0.24 ± 0.09
M6	GS	157 ± 39*	15 ± 5	140 ± 29*§	98 ± 1	37 ± 8	37.4 ± 0.9	0.25 ± 0.11
	GB	149 ± 31*	12 ± 3*	112 ± 16§	98 ± 3	35 ± 11*	37.5 ± 0.6*	0.24 ± 0.09
M7	GS	148 ± 36	19 ± 4§	123 ± 30§	99 ± 1	36 ± 10	37.5 ± 0.9	0.27 ± 0.11
	GB	128 ± 45	13 ± 5*§	92 ± 16§	99 ± 2	40 ± 8*	37.6 ± 0.7*	0.2 ± 0.11

No differences were observed between the two groups at any moment or between M0 and other moments. The table includes heart rate (HR), in beats per minute; respiratory rate (RR), in breaths per minute; systolic arterial pressure (SAP), in mmHg; peripheral oxygen saturation (SpO_2_), as a percentage; end-tidal carbon dioxide concentration (EtCO_2_), in mmHg; esophageal temperature (T^o^C), in degrees Celsius; and propofol infusion rate (Propofol), in mg/kg/min. The data are presented across the following moments: M0 (after anesthetic induction and stabilization), M1 (5 min after completion of the ESP block), M2 (15 minutes after the block), M3 (skin incision), M4 (clamping of the right ovarian pedicle), M5 (clamping of the left ovarian pedicle), M6 (ligation of the cervix), and M7 (celiorrhaphy). (*) indicates a statistically significant difference from M0; (§) indicates a statistically significant difference between groups at the same time point. Differences were considered statistically significant at *p* ≤ 0.05

Postoperative pain scores assessed using the UNESP–Botucatu Multidimensional Pain Scale are presented in Table 3. In the bupivacaine group (GB), pain scale scores differed significantly from baseline at T2 (*p* = 0.0020). In the saline group, significant increases in pain scores were observed at T2 and persisted at T4 (*p* = 0.0002). Total pain scores differed from baseline at T2 in the GB group (*p* < 0.0001) and at both T2 and T4 in the GS group (*p* = 0.0001). All cats that reached the predefined rescue analgesia threshold received buprenorphine and were subsequently withdrawn from the study, resulting in no animals remaining for evaluation beyond 4 h postoperatively.

**Table 3:** Median (interquartile range) postoperative pain and sedation scores in cats undergoing elective ovariohysterectomy following an erector spinae plane block with 0.25% bupivacaine (GB) or saline solution (GS) administered at 0.5 mL/kg per injection site. Rescue analgesia was based exclusively on the UNESP–Botucatu scale: cats with a total score ≥ 4

Times	Group	Pain	Sedation	Rescues	Number of animals
T0	GS	0 [0–0]	0 [0–0.75]	0	8
	GB	0 [0–0.25]	2.5 [0–3]	0	8
T2	GS	4 [2.75–5.75]*	5.5 [4.5–7.5]*	5	8
	GB	5 [4,75 − 6]*	4.5 [3–5]	7	8
T4	GS	4 [4–4]*	8 [7.5–8]*	3	3
	GB	4 [4–4]	3 [3–3]	1	1
T6	GS	N/a	N/a	N/a	0
	GB	N/a	N/a	N/a	0
T8	GS	N/a	N/a	N/a	0
	GB	N/a	N/a	N/a	0
T12	GS	N/a	N/a	N/a	0
	GB	N/a	N/a	N/a	0
T24	GS	N/a	N/a	N/a	0
	GB	N/a	N/a	N/a	0

Time points included T0 (before administration of preanesthetic medication) and 2 (T2), 4 (T4), 6 (T6), 8 (T8), 12 (T12), and 24 (T24) hours after the end of surgery. “Number of animals” refers to the number of cats evaluated at each time point, excluding individuals that had already received rescue analgesia at earlier assessments. Animals requiring rescue analgesia were withdrawn from subsequent postoperative evaluations. “N/a” indicates that no animals remained eligible for assessment at that time point and, therefore, no data were available. (*) indicates a statistically significant difference from T0

**Fig. 4:**
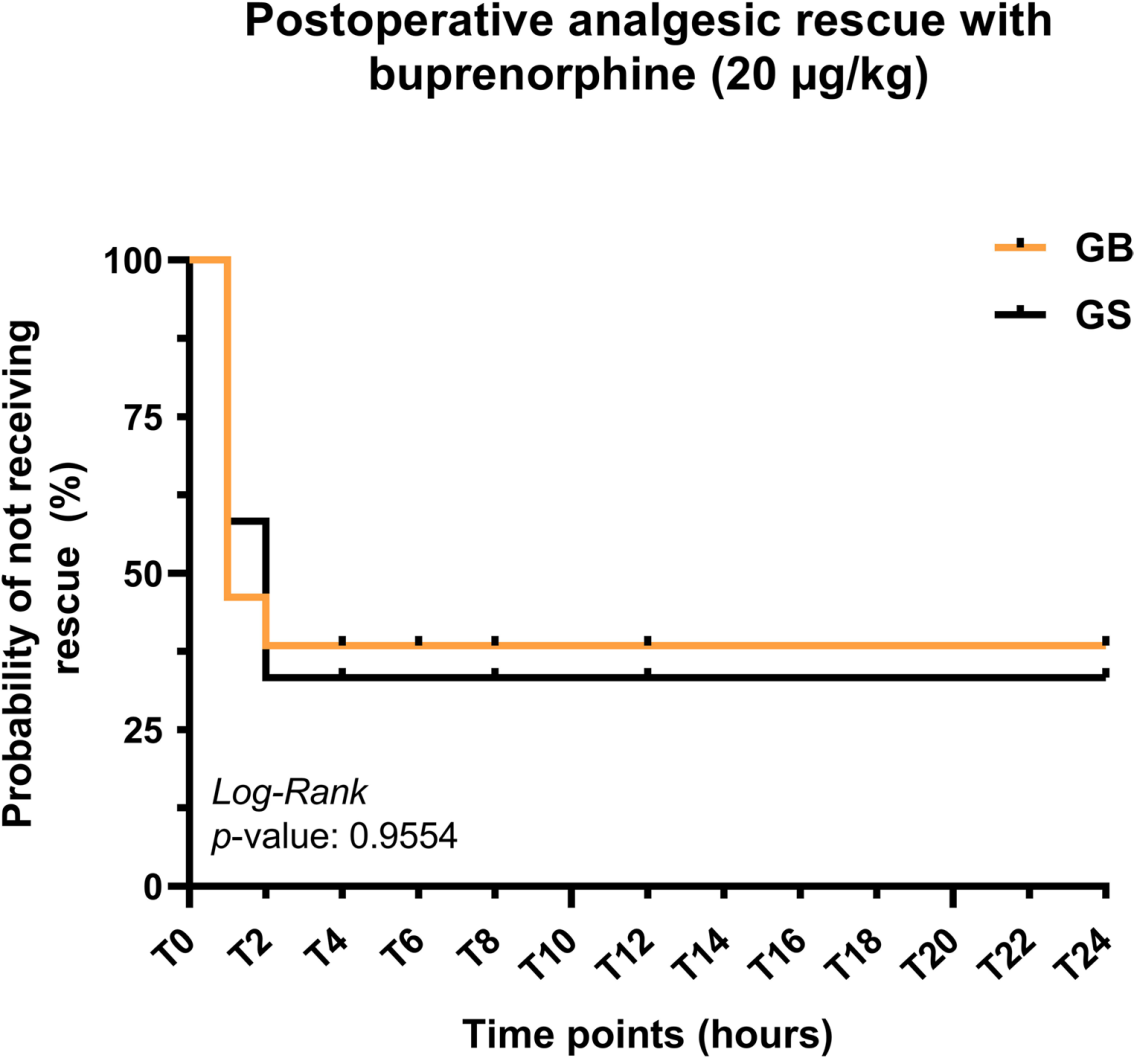
Kaplan–Meier curve illustrating the probability of remaining free from postoperative rescue analgesia with buprenorphine 20 ug/kg; in cats anesthetized with propofol and undergoing elective ovariohysterectomy after an ultrasound-guided erector spinae plane block with 0.25% bupivacaine (GB) or saline solution (GS)

Postoperative rescue analgesia with buprenorphine was required in both groups within the first postoperative hours. In the bupivacaine group (GB), seven cats required rescue analgesia at 2 h after extubation, and one additional cat required rescue at 4 h. In the GS, five cats required rescue analgesia at 2 h and three cats at 4 h postoperatively. Kaplan–Meier survival analysis demonstrated that most rescue analgesic interventions occurred at the 2 h postoperative time point in both groups, with a comparable temporal distribution of rescue requirements thereafter and no clear divergence between the survival curves (Fig. 4). These results indicate insufficient postoperative analgesic control following elective ovariohysterectomy in cats under the protocols evaluated.

Sedation scores are presented in Table 3. Total sedation scores increased significantly following postoperative analgesic rescue with buprenorphine, with higher scores observed shortly after rescue administration and a subsequent reduction over time. Total sedation scores increased significantly following postoperative rescue analgesia with buprenorphine compared with baseline (T0).

## Discussion

All cats completed the study successfully, and no animal lost follow up. The primary hypothesis that the use of an ultrasound-guided erector spinae plane (ESP) block with bupivacaine would reduce the requirement for intraoperative and postoperative rescue analgesia compared with saline was only partially supported. The results suggest that integrating the ESP block into a multimodal anesthetic protocol contributed to reduced intraoperative opioid requirements and modulation of autonomic responses associated with surgical nociception. The analgesic effects of local anesthetics result from inhibition of voltage-gated sodium channels, preventing the propagation of nociceptive impulses along sensory nerve fibers (Catterall and Swanson, 2015). The second hypothesis was not confirmed, as a consistent postoperative analgesic benefit was not demonstrated. Overall, these results suggest that, under the conditions of the present study, the analgesic contribution of the ESP block was predominantly limited to the intraoperative period, with minimal extension into postoperative analgesia. One possible explanation is the dilution of bupivacaine, which may have reduced its duration of action (Fenten et al. 2015; Eng et al. 2014; Grubb and Lobprise, 2020), thereby contributing to adequate intraoperative analgesia without prolonged postoperative benefit. Additionally, postoperative pain assessment may have been influenced by overlap between sedation-related behavioral changes and nociceptive parameters included in validated pain scales (Girard et al., 2010; Benito et al., 2017; Schmitz et al., 2026). Comparable findings have been reported in dogs, in which the ESP block performed with bupivacaine 0.5% reduced intraoperative analgesic requirements for ovariohysterectomy in bitches without demonstrating a sustained postoperative analgesic benefit (Schmitz et al., 2026). Propofol infusion was titrated according to predefined anesthetic planes and clinical criteria (Ribeiro et al., 2009), and the ESP block with bupivacaine was not associated with a reduction in hypnotic requirements, indicating the absence of a propofol-sparing effect under the conditions of this protocol. This finding is not unexpected, as interfascial plane blocks are primarily intended to attenuate peripheral nociceptive input rather than directly modulate cortical hypnosis. Comparable results were reported by Degani et al. (2023), who evaluated different concentrations of ropivacaine for quadratus lumborum block in bitches undergoing ovariectomy and observed no reduction in hypnotic agent consumption; despite effective intraoperative analgesia, when compared with a fentanyl-based protocol using sevoflurane anesthesia. Propofol produces dose-dependent cardiovascular and respiratory effects through mechanisms including calcium channel modulation and nitric oxide–mediated vasodilation (Larsen et al., 2007; Saugel et al., 2022); however, in cats, slow titration within clinically recommended infusion ranges has been shown to preserve acceptable hemodynamic stability (Robertson et al., 2018). To date, no clinical studies have specifically evaluated propofol-sparing effects of the ESP block in cats, limiting direct comparison with the present findings. Therefore, the intraoperative benefits observed with the ESP block are more likely attributable to modulation of nociceptive transmission rather than a reduction in intravenous anesthetic demand, as propofol primarily exerts its anesthetic effects through potentiation of γ-aminobutyric acid (GABA)–mediated inhibitory neurotransmission (Ying and Goldstein, 2005). Notably, no animals developed clinical evidence of local anesthetic systemic toxicity at the concentration and volume of bupivacaine administered. The hypocapnia observed at the beginning of the procedure may be associated with several factors, including the small body size of the animals, which may increase susceptibility to ventilatory variability, and an anesthetic plane that was not yet fully stabilized, potentially promoting transient hyperventilation (Robertson et al., 2018). Another possible explanation involves species-related differences in carbon dioxide values, as venous blood gas analyses in cats have demonstrated lower partial pressure of carbon dioxide values (median 30 mmHg; range 28–32 mmHg) compared with dogs (median 37 mmHg; range 32–43 mmHg) (Silverstein and Hopper, 2015). These physiological differences may partially explain the lower EtCO₂ values observed in feline patients. Assessment of autonomic responses to intraoperative nociception is ideally performed using invasive arterial blood pressure monitoring, the gold standard due to its accuracy and continuous waveform analysis. However, arterial catheterization increases procedural complexity, may be influenced by dexmedetomidine-induced vasoconstriction, and carries potential risks in client-owned cats, particularly in small patients. Oscillometric monitoring, although less precise and affected by cuff size and peripheral perfusion, is widely used in veterinary anesthesia because it is non-invasive and reliable for detecting relative hemodynamic changes during surgical stimulation, as reported in similar feline studies (Comassetto et al., 2025; Conterno et al., 2025). In the present study, ESP block with bupivacaine was associated with improved intraoperative analgesic stability when incorporated into a multimodal anesthetic protocol. Attenuation of autonomic responses during periods of increased surgical stimulation is consistent with previous veterinary reports supporting the clinical application of the ESP block for perioperative pain management in dogs, particularly in thoracic and spinal procedures (Zannin et al., 2020; Bartholomew and Ferreira, 2021; Gomez Fernandez et al., 2021; Portela et al., 2021). These effects are likely related to partial blockade of the dorsal and ventral rami of the spinal nerves, as well as to potential diffusion of the injectate toward paravertebral and sympathetic structures, as demonstrated in anatomical and cadaveric investigations in dogs (Ferreira et al., 2019; Medina-Serra et al., 2021; Herrera-Linares et al., 2024). In addition, it has been proposed that injectate administered within the erector spinae plane may spread to contiguous anatomical compartments, thereby promoting anesthesia of the ventral branches of the spinal nerves in human patients (Chin & El-Boghdadly, 2021), a mechanism shown to provide effective thoracic analgesia, possibly through involvement of these ventral branches and adjacent neural structures (Forero et al., 2016; Chin et al., 2019). Consequently, the clinical effectiveness of the ESP block appears to depend not only on local anesthetic distribution within the erector spinae plane itself, but also on the extent of its spread to neighboring fascial, paravertebral, and neural spaces. Its use in regional anesthetic techniques has been associated with reduced opioid requirements and improved perioperative cardiovascular stability in veterinary patients (Ferreira et al., 2019; Viilmann et al., 2022). In cats, however, species-specific pharmacokinetic characteristics, particularly limited hepatic glucuronidation, require cautious dosing to minimize the risk of systemic accumulation and toxicity (Garbin et al., 2022; Grubb and Lobprise, 2020). Although the long duration of action of bupivacaine makes it suitable for procedures such as ovariohysterectomy, dilution to achieve the volumes required for interfascial plane blocks may reduce the duration and consistency of postoperative analgesia (Fenten et al. 2015; Eng et al. 2014). More diluted local anesthetic solutions may produce shorter sensory and motor blockade compared with higher concentrations of the same drug, likely due to reduced availability of drug molecules to sustain sodium channel blockade at the nerve membrane (Eng et al., 2014). Similar observations have been described in dogs, in which an ESP block performed with 0.5% bupivacaine decreased intraoperative analgesic requirements during ovariohysterectomy in bitches but did not result in sustained postoperative analgesia (Schmitz et al., 2026). Despite favorable intraoperative effects, the ESP block alone was insufficient to provide complete postoperative analgesia in all animals. This finding aligns with previous reports suggesting that interfascial plane blocks may offer limited and variable visceral analgesia, particularly when used as a single regional technique in humans (Yayik et al., 2019Kwon et al., 2020). In cats, an ultrasound-guided transversus abdominis plane (TAP) block with bupivacaine has been reported to improve postoperative pain control when incorporated into a multimodal analgesic protocol, underscoring the importance of combining regional techniques with systemic analgesics to achieve effective postoperative analgesia (Skouropoulou et al., 2018).

The limited involvement of the thoracic splanchnic nerves when the erector spinae plane block is performed at a lumbar level may partly account for the persistence of postoperative pain following ovarian manipulation. In cats, ovarian visceral afferent innervation originates predominantly from thoracic spinal segments T8 to T12, which may not be adequately covered by a lumbar ESP block, thereby limiting visceral analgesia and contributing to residual postoperative pain (Fink and Schofield, 1971). In contrast, Cavalcanti et al. (2022) performed ESP injections in dogs at the T12 level, targeting the transverse or mammillary processes, which may favor greater cranial spread and broader neural coverage. Differences in analgesic outcomes between that study and the present findings may therefore be related to variations in injection level, injectate volume, and local anesthetic concentration. More diluted local anesthetic solutions may produce shorter sensory and motor blockade compared with higher concentrations of the same drug (Eng et al., 2014). These observations highlight the mammillary process as a relevant anatomical landmark for ultrasound-guided ESP block performance in the thoracolumbar region. When appropriately targeted, the ESP block has been suggested as an effective analgesic technique for procedures such as hemilaminectomy in dogs, supporting its role in controlling somatic and paraspinal nociception (Zannin et al., 2020; Portela et al., 2021).

In cats, ESP injections have been described in the lumbar region. A clinical report showed perioperative analgesia for spinal surgery (Alza Salvatierra et al. 2021). In addition, a recent cadaveric study demonstrated a volume-dependent spread of injectate following lumbar ESP injection, with predominant distribution to the dorsal branches of the spinal nerves (DBSN), and occasional extension to the ventral branches of the spinal nerves (VBSN), intercostal muscles, and hypaxial musculature (Nobre et al., 2025). To date, however, the anatomical spread and potential visceral coverage of thoracic ESP injections in cats have not been investigated, limiting definitive conclusions regarding the capacity of this technique to provide consistent visceral analgesia in feline abdominal surgery.

Postoperative pain assessment in cats remains inherently challenging due to their subtle behavioral responses and tendency to mask discomfort (Steagall and Monteiro, 2019). The combined use of the UNESP-Botucatu Multidimensional Pain Scale and the Feline Grimace Scale allowed a more comprehensive evaluation by integrating both behavioral and facial expression–based indicators of pain (Brondani et al., 2013; Evangelista et al., 2019). In the present study, both pain assessment scales were sensitive in detecting postoperative pain. Importantly, when rescue analgesia was indicated by one scale, it was consistently supported by the other, demonstrating concordance between the instruments. Nevertheless, the Feline Grimace Scale appeared to be more sensitive in guiding rescue analgesia decisions, in agreement with recent reports supporting its validity and clinical utility for feline pain assessment (Cheng et al., 2023; Marangoni et al., 2025).

Sedation scores in the postoperative period should be interpreted cautiously, as residual anesthetic and opioid effects may overlap with behavioral indicators of pain. Although buprenorphine is a partial µ-opioid receptor agonist with a comparatively lower sedative profile, it may still induce central nervous system depression and behavioral changes in cats (Steagall and Monteiro, 2019). Opioids such as fentanyl and buprenorphine may induce dose-dependent reductions in activity, alertness, and responsiveness, which can confound pain scoring (Steagall and Monteiro, 2019). Conversely, cats experiencing pain may also exhibit decreased interaction and spontaneous movement, potentially mimicking sedative effects (Steagall and Monteiro, 2019).

Several limitations of the present study should be acknowledged. Dilution of bupivacaine to achieve the target injection volume may have reduced the duration of postoperative analgesia, potentially limiting its sustained clinical effect (Grubb and Lobprise, 2020). In addition, although the sample size was considered adequate based on prior methodological frameworks, the relatively small number of animals may restrict the generalizability and external validity of the findings. The absence of invasive arterial blood pressure monitoring represents an additional limitation, as direct arterial measurements may provide greater accuracy in the assessment of intraoperative hemodynamic responses. Furthermore, advanced objective monitoring tools, such as parasympathetic tone index and surgical pleth index, were not evaluated and could have contributed to a more comprehensive assessment of intraoperative nociception. Finally, the use of validated pain and sedation scales, postoperative pain assessment in cats remains inherently subjective and dependent on behavioral interpretation, which may introduce variability and limit the precision of outcome measurements.

## Conclusion

In conclusion, the erector spinae plane block with bupivacaine may provide beneficial intraoperative opioid-sparing effects when incorporated into a multimodal anesthetic protocol for feline ovariohysterectomy. However, when applied as a sole regional technique, it did not provide adequate postoperative analgesia. These findings support the role of the ESP block as an adjunct rather than a standalone technique and reinforce the need for comprehensive multimodal analgesic strategies in feline abdominal surgery.

## Data Availability

The datasets generated and/or analyzed during the current study are available from the supervising authors (F.C. and N.O.) upon reasonable request.
